# Identification of microstructures critically affecting material properties using machine learning framework based on metallurgists’ thinking process

**DOI:** 10.1038/s41598-022-17614-0

**Published:** 2022-08-20

**Authors:** Satoshi Noguchi, Hui Wang, Junya Inoue

**Affiliations:** 1 Department of Advanced Interdisciplinary Studies, The University of Tokyo, 4-6-1 Komaba, Meguro, Tokyo 153-8904 Japan; 2 Institute for Industrial Science, The University of Tokyo, 5-1-5 Kashiwanoha, Kashiwa, Chiba 277-0082 Japan; 3 Department of Materials Engineering, The University of Tokyo, 7-3-1 Hongo, Bunkyo, Tokyo 113-8656 Japan; 4 Research Center for Advanced Science and Technology, The University of Tokyo, 4-6-1 Komaba, Meguro, Tokyo 153-8904 Japan

**Keywords:** Metals and alloys, Structural materials, Mechanical properties

## Abstract

In materials science, machine learning has been intensively researched and used in various applications. However, it is still far from achieving intelligence comparable to that of human experts in terms of creativity and explainability. In this paper, we investigate whether machine learning can acquire explainable knowledge without directly introducing problem-specific information such as explicit physical mechanisms. In particular, a potential of machine learning to obtain the capability to identify a part of material structures that critically affects a physical property without human prior knowledge is mainly discussed. The guide for constructing the machine learning framework adopted in this paper is to imitate human researchers’ process of thinking in the interpretation and development of materials. Our framework was applied to the optimization of structures of artificial dual-phase steels in terms of a fracture property. A comparison of results of the framework with those of numerical simulation based on governing physical laws demonstrated the potential of our framework for the identification of a part of microstructures critically affecting the target property. Consequently, this implies that our framework can implicitly acquire an intuition in a similar way that human researchers empirically attain the general strategy for material design consistent with the physical background.

## Introduction

In the field of computational materials science, machine learning has been intensively investigated and used in a wide range of applications^1–8^. Machine learning methodologies can extract hidden patterns or capture implicit relations from a tremendous amount of data, such as material micrographs and material structure-property data. However, they remain far from achieving intelligence comparable to human intelligence in terms of creativity and explainability, which are essential in the field of material design^9–11^. It is not clear how these methodologies capture the material or physical background of the extracted patterns; thus, the captured knowledge is not immediately applicable to other general cases, which becomes a crucial issue when applying machine learning to material design. The complexity and the lack of clarity of the mechanisms underlying the process of generating material microstructures require improved explainability that can provide a general guide for designing materials. On the other hand, humans historically have been able to acquire empirical knowledge with which a general strategy of material design can be derived from much less amount of experimental data; that is, human experts seem to obtain an *intuition* for material design from their experiences. This fact motivates us to develop a machine learning framework based on human experts’ train of thoughts for material design.

A fundamental idea that metallurgists share in common is that material microstructures are composed of finite kinds of dissimilar phases or small-scale microstructures. Since individual small-scale microstructures develop competitively with completely different formation kinetics, they are supposed to have totally different geometrical features while maintaining certain spatial orders depending on process conditions such as cooling rate and holding temperature^12–15^. On the basis of this understanding, our group successfully demonstrated previously that a convolutional neural network (CNN) provides an efficient route to extract a finite number of geometrical features representing each small-scale microstructure, and we proposed an unsupervised machine learning framework for the segmentation of steel microstructures^16^. The concept was further extended to automatically generate material microstructures from process parameters^17^. In this framework, we adopted a vector quantized variational autoencoder (VQVAE) to extract a certain number of characteristic geometrical features from optical micrographs of steel microstructures and a pixel convolutional neural network (PixelCNN) to reveal spatial orders of small-scale microstructures as a function of process parameters.

In the present paper, we demonstrate the capability of the proposed machine learning framework consistent with metallurgists’ process of thinking to acquire physically explainable knowledge rather than simple segmentation or autogeneration problems, hoping to get closer to the train of thoughts that metallurgists implicitly attain the know-how to design materials. The validity of this framework is demonstrated in the context of the microstructure optimization of fracture elongation of dual-phase steels.

The following aspects are covered in this paper. (i) The consistency between the machine learning framework composed of VQVAE and PixelCNN and metallurgists’ train of thoughts is explained. (ii) As an example of material design, a structure optimization problem for dual-phase materials concerning a fracture property is analyzed using a dataset numerically computed using the Gurson–Tvergaard–Needleman (GTN) fracture model^18,19^. The results indicate that the framework can capture the physical relationship between material microstructures and the target property in various cases. (iii) To clarify the knowledge captured by the present framework, we seek to identify a part of microstructures that critically affects the fracture property by calculating the gradient of material microstructures with respect to the target property based on the machine learning framework composed of VQVAE and PixelCNN. If the machine learning framework correctly captures the correlation between the geometry of the material microstructures and the fracture strain, this gradient is supposed to show relatively high values for the area that strongly influences the fracture strain. This is based on the assumption that human experts unconsciously consider the *sensitivity* of material structures to a change in target property to optimize microstructures. The hot spot identified by the present framework corresponds at an acceptable level to those clarified in the numerical simulation based on the explicit physical model. This implies that our framework can predict a part of microstructures that strongly influences a physical property in a similar way that human experts intuitionally capture it. In this sense, we show in this paper that imitating experts’ train of thoughts, which is based on an intense consideration with a deep understanding of the physical background, could be a guide for designing a machine learning framework that will have a potential to capture critical points as human experts do with much higher efficiency and explainability.

## Methodology

### Implementing metallurgists’ process of thinking using VQVAE and PixelCNN

A guide for the construction of the machine learning framework that we have adopted is to imitate metallurgists’ train of thoughts when designing metallic materials. As shown in Introduction, metallurgists interpret that material microstructures are composed of finite kinds of characteristic small-scale microstructures which develop with completely different formation kinetics. For example, steel alloys exhibit a wide range of mechanical properties owing to the presence of various internal structures such as ferrite, pearlite, bainite, and martensite. These internal structures in steel alloys critically affecting many essential properties are determined by dynamical phase transformation processes during heat treatments. The phase transformation processes in steel alloys can be roughly divided into two categories: diffusional transformation based on the diffusion of atoms in alloys and displacive transformation based on the deformation of the original atomic pattern into a new crystal structure^14^. Basically, ferrite and pearlite are considered to originate from diffusional transformation, whereas bainite and martensite are understood to be generated by displacive transformation^13–15^. In addition, pearlite and ferrite should also be distinguished owing to the differences in their formation processes and geometrical characteristics^15^, and bainite and martensite are recognized to have critically different nucleation processes as well^13^. From the above, steel alloys are commonly interpreted to be composed of various characteristic structures with completely different geometrical configurations due to qualitatively different physical backgrounds. Moreover, it should also be recognized that the formation processes of the above characteristic steel microstructures are dynamical. In other words, individual characteristic microstructures are dynamically determined through their mutual interactions. Thus, the resultant arrangement of characteristic microstructures is supposed to have some spatial order. This can be a hint for the choice of machine learning frameworks.

**Figure 1:**
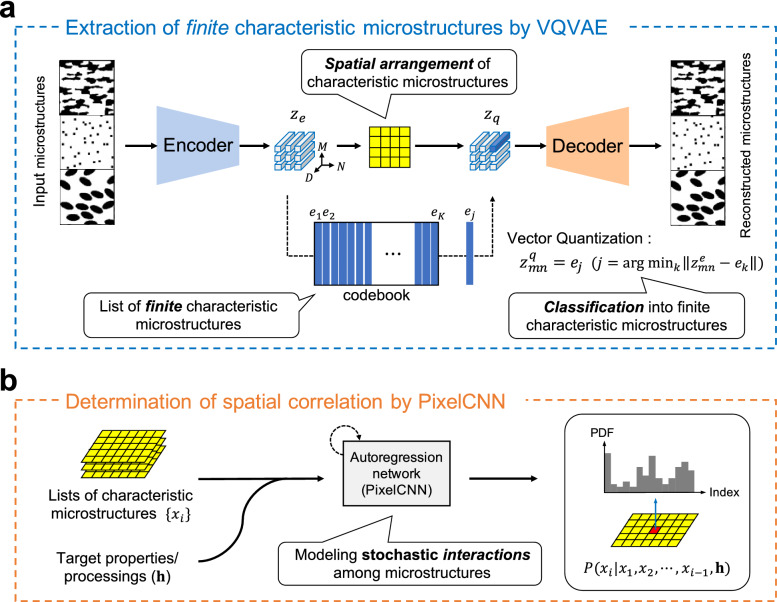
Machine-learning-based computational framework for characterization and generation of material microstructures. This framework was strongly motivated by metallurgists’ thought process in interpreting material structures. (**a**) Extraction of *finite* characteristic microstructures by VQVAE. (**b**) Determination of spatial correlation among the extracted finite characteristic microstructures by PixelCNN. PDF stands for probability density function. This architecture is suitable for expressing metallurgists’ thought process leading to the interepretation that material structures are composed of finite kinds of characteristic fundamental elements with completely different geometrical features and that the generation of material structures stems from interactions among the elements.

From the above metallurgists’ interpretation of material microstructures, a machine learning framework to represent the microstructure generation should have two essential capabilities: (i) to extract various *qualitatively different* characteristic microstructures that form a target metallic material and (ii) to determine some spatial order among the extracted characteristic microstructures. To implement these components, in this paper, we adopt VQVAE^20^ for the extraction of characteristic microstructures and PixelCNN^21,22^ for the determination of the spatial order among them.

Figure 1 illustrates the schematic of our framework. As shown in Fig. 1a, VQVAE includes two important functional components that reflect metallurgists’ process of thinking: the codebook and vector quantization (VQ). The codebook is defined as a set of candidates for discrete latent vectors describing features of local regions in input images, which is refined and optimized by training. Thus, the codebook after training corresponds to a list of structural basic components included in input metallic materials. In the cases of dual-phase steels, ferrite/martensite grains or grain boundaries are considered as examples of the basic components. Also, the VQ is the procedure in which outputs by the CNN-based encoder are replaced by a set of latent vectors included in the codebook. This procedure can be regarded as the classification of small-scale microstructures in input material images into the characteristic microstructures included in the codebook. Importantly, since VQVAE has the discrete latent vectors originating from different distributions independent of each other, it can extract a list of *qualitatively different* characteristic microstructures that form the material structures. In contrast, the variational autoencoder (VAE)^23^ and generative adversarial networks (GANs)^24^, which are the two major algorithms of generative models, perform a generative process based on the latent vectors stemming from one continuous distribution. Thus, it is difficult to extract qualitatively different geometrical structures using VAE or GANs. In this sense, we consider that VQVAE is a better choice for extracting characteristic material structures with completely different physical backgrounds. This can be rephrased that the architecture of VQVAE is more consistent with our interpretation of material microstructures than other networks. Owing to these two essential components, VQVAE can deconstruct the material structures into the spatially arranged characteristic elements included in the codebook, in a similar way that metallurgists identify fundamental structures and deconstruct material structures into a set of fundamental elements.

The other network forming our framework is PixelCNN^17,21,22^. As shown in Fig. 1b, PixelCNN is designed to capture the spatial order in the material structures. In particular, it is implemented to model the joint distribution of characteristic microstructures over a material structure as the following product of conditional distributions for the $n\times n$ list of characteristic microstructures:

1 $$\begin{aligned} P(X| {{\textbf {h}}})=P(x_1|{{\textbf {h}}})\prod _{i=2}^{n^2}P(x_i|x_1,\ldots ,x_{i-1}, {{\textbf {h}}}), \end{aligned}$$

where *X* represents an input material structure, $x_i$ is a characteristic microstructure included in the input material structure *X*, and ${{\textbf {h}}}$ is the vector of the given conditions, such as material properties. The ordering of the pixel dependences is from left to right and from top to bottom. This expresses the stochastic spatial arrangement of the small-scale microstructures over a material structure resulting from competitive generation processes of material microstructures. In this sense, this implementation is also considered to be consistent with our assumption that material microstructures are supposed to have some spatial order because of the dynamical process of microstructure generation. At the same time, PixelCNN captures correlations between material structures and a given material property, which results in the determination of the structure-property relationship. This was also discussed in^17^.

Consequently, the important and valuable characteristic of our framework is its consistency with metallurgists’ interpretation of material microstructures, due to the identification of fundamental structures included in target materials by VQVAE and the determination of the spatial order among them by PixelCNN.

### Details of network architecture

The encoder and decoder included in VQVAE shown in Fig. 1a are implemented by CNN. Also, the codebook is composed *K* *D*-dimensional latent vectors. The CNN-based encoder outputs $z^e\in \mathbb {R}^{M\times N \times D}$, which is a set of $M\times N$ *D*-dimensional vectors. Then, each *D*-dimensional vector included in $z^e$ is replaced with a nearest vector in the codebook. The decoder regenerates the input image from a set of replaced latent vectors $z^q\in \mathbb {R}^{M\times N \times D}$. The details of network was also discussed in^17^.

The error function of VQVAE is composed of three error terms, reconstruction error, codebook error, and commitment error^17,20^, as the first, second and third term in the following equation, respectively,

2 $$\begin{aligned} \mathcal {L}_{VQVAE}= & {} ||x-\hat{x}||_2+||\phi _{sg}(z^e)-z^q||_2+\beta ||z^e-\phi _{sg}(z^q)||_2, \end{aligned}$$

where $\phi _{sg}$ is the stop-gradient operator and $\beta$ is the weight for adjusting the influence of the commitment error. The codebook error is used for making chosen vectors included in codebook $z^q$ approach the corresponding *D*-dimensional vectors in $z^e$, while the commitment error is applied for making vectors in $z^e$ close to the selected vectors in codebook $z^q$ with respect to $L^2$-distance. $\phi _{sg}$ is introduced so that $z^e$ and $z^q$ can approach each other alternately.

As a result of training, VQVAE can extract two-dimensional index lists corresponding to spatial arrangement of characteristic microstructures included in the input image. The index lists are represented as an $(M\times N)$-dimensional integer matrix. In other words, input images can be converted into the index lists using the trained encoder included in VQVAE. Each pixel in the index lists has *K* possible values. Using PixelCNN, we capature the spatial correlation of each pixel in the index lists defined as Eq. (1). The ordering of the pixel dependences is left to right and top to bottom. The procedure to achieve this dependences by CNN can be found in^17,21,22^. The inputs of PixelCNN are the index lists and the conditions such as fracture strain and/or strength. Then, PixelCNN is trained using the cross-entropy loss function for the expectation of the inference to be identical to the inputted true index list.

For the analysis reported in this paper, we set *M*, *N*, and *D* as 16, 16, and 128, respectively. Also, the number of latent vectors included in codebook *K* is set as 512 and $\beta$ is set as 1.0. The number of epochs were 1000 for both of VQVAE and PixelCNN. In addition, the number of convolutional layers of PixelCNN is 15 in this paper. We trained VQVAE and PixelCNN using all of 3824 prepared artificial microstrucrture images. The other details of implementation such as filter sizes in CNN networks is publically available on Github repository shown in Code availability.

### Experiment and numerical study for preparing training dataset

The ductile fracture of dual-phase steels is due to the void formation, growth and coalescence^25^. In this study, void volume fraction (VVF) was used as the identifier of fracture. Numerical studies were used to prepare the dataset of artificial microstructures for machine learning, while the experiments were conducted to calibrate the material parameters of the numerical simulation.

The used material in this study was DP590. To obtain the fracture strain, uniaxial tension testing was performed to the as-received material using Instron 4204. The tension testing was performed at ambient temperature and with an initial strain rate of 0.001/s. The load-displacement curve was recorded.

**Figure 2:**
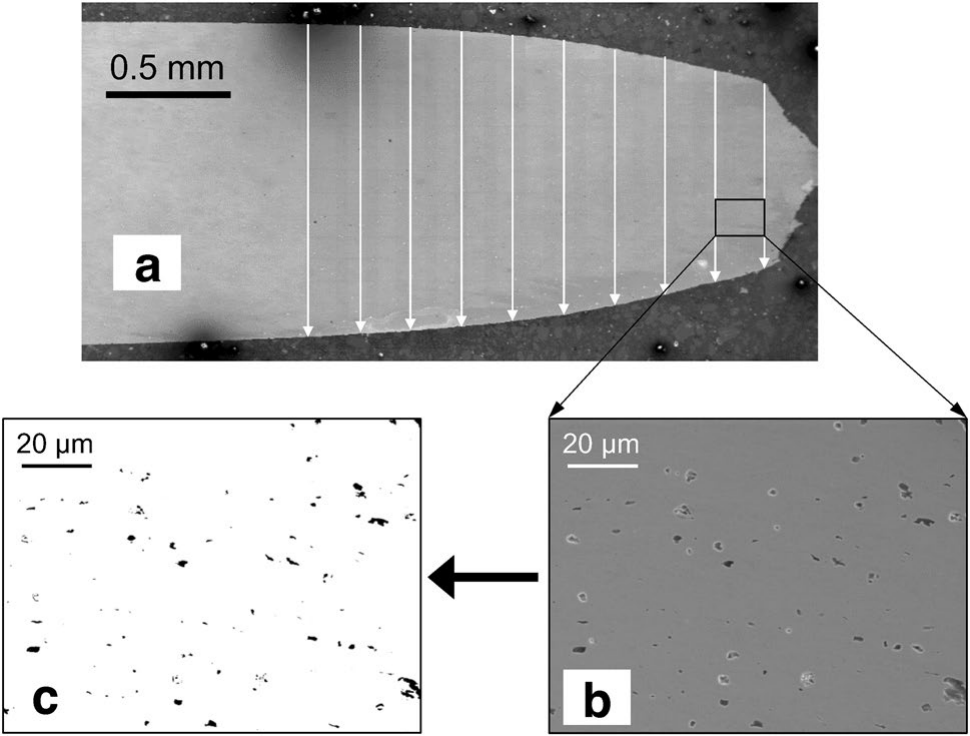
Procedure of experiment for measuring the void volume fraction. (**a**) SEM scanned regions in the necked region. (**b**) A typical SEM image. (**c**) Identified voids using ImageJ.

After the fracture, the voids in the necked regions were observed using a scanning electron microscopy (SEM), as shown in Fig. 2a. A standard metallographic procedure was used to prepare the SEM samples. SEM measurements were carried out by field-emission SEM (JEOL, JSM-7200F) under an acceleration voltage of 15.0 kV. The SEM characterization was along the whole thickness (Fig. 2a), and covered from the necked to fractured regions. ImageJ was adopted to identify the voids (Fig. 2c), and the VVF was evaluated according to the area of void within the area of the observation (Fig. 2b). This VVF was used as the threshold to determine the fracture strain in numerical simulations.

**Figure 3:**
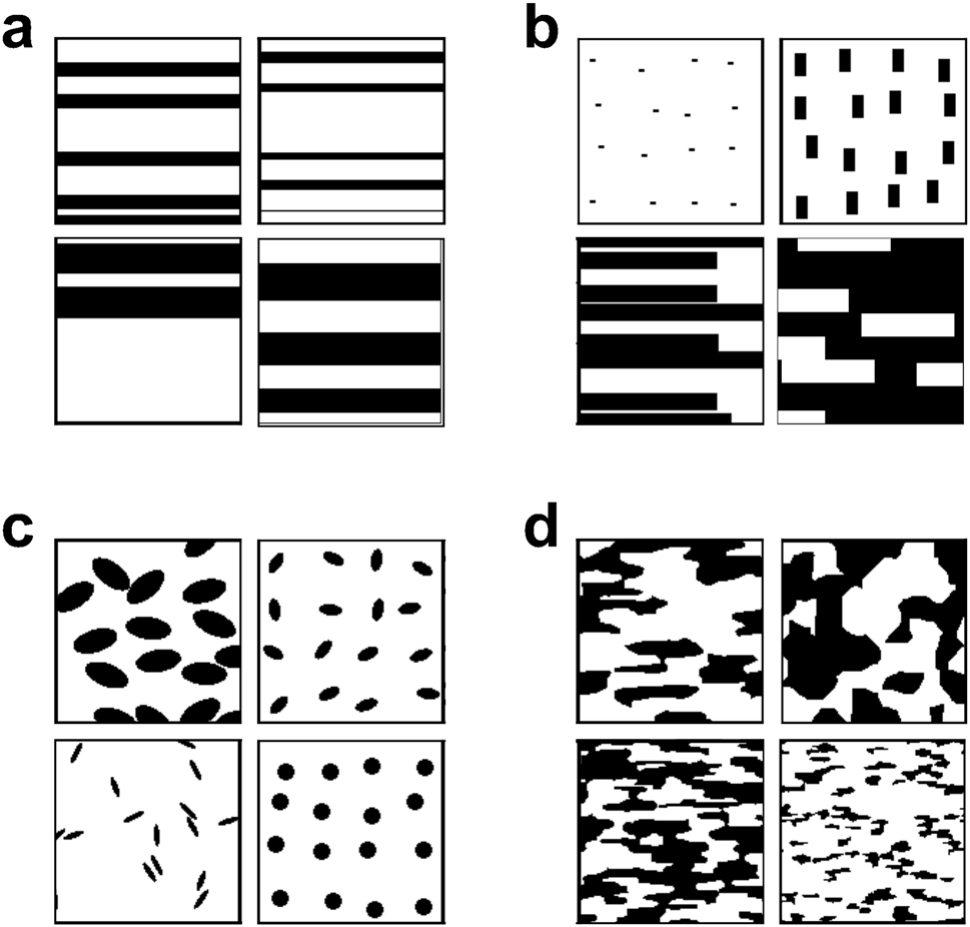
Examples of artificial dual-phase microstructures used for training. Black and white pixels respectively correspond to the hard phase (martensite) and soft phase (ferrite). The size of microstructure images is $128\times 128$ pixels. The dataset can be divided into four major categories. (**a**) Laminated microstructures. This category only has completely laminated microstructures. (**b**) Microstructures composed of rectangular martensite grains. This category includes partially laminated structures, such as these shown in the lower left panel. (**c**) Microstructures composed of elliptical martensite grains. (**d**) The random microstructures.

Various types of two-dimensional dual-phase ferrite-martensite microstructures were artificially prepared using open-source software Dream3D^25^ and commercial software Matlab. Irregular morphologies (approximating real dual-phase microstructures) were generated using Dream3D, and the martensite volume fraction, aspect ratio, and grain size were varied. In contrast, regular shapes (laminated, ellipse, and rectangle) of martensitic grains were generated using Matlab, and the martensite volume fraction, aspect ratio, grain size, grain direction, and grain distribution were varied. The distribution of martensitic grains incrementally evolved from regular to random. Examples of microstructures are shown in Fig. 3. The total number of microstructures was 3824.

Finite element method (FEM) was utilized to predict the fracture of artificial microstructures^26^. In the simulation, the representative volume element (RVE) was meshed into 128$\times$128 elements (pixels), and a phase, either ferrite or martensite, was assigned to each element. Two-dimensional plane strain conditions were assumed, and four-node bilinear elements with reduced integration were used. Tension was applied to the left and right sides of the sample, and the load and displacement were recorded. Abaqus Ver.2018 was used for numerical simulations.

Gurson–Tvergaard–Needleman (GTN) model, a ductile damage model, was introduced into the FEM simulations^18,19^. The experimentally obtained relationship between engineering strain and accumulated VVF was used to calibrate the material parameters of the GTN model. After the simulations, the average VVF over the entire sample was calculated after each displacement increment, and then fracture strain was determined for each microstructure.

## Results and discussion

### Analysis of structure optimization problem of dual-phase materials

**Figure 4:**
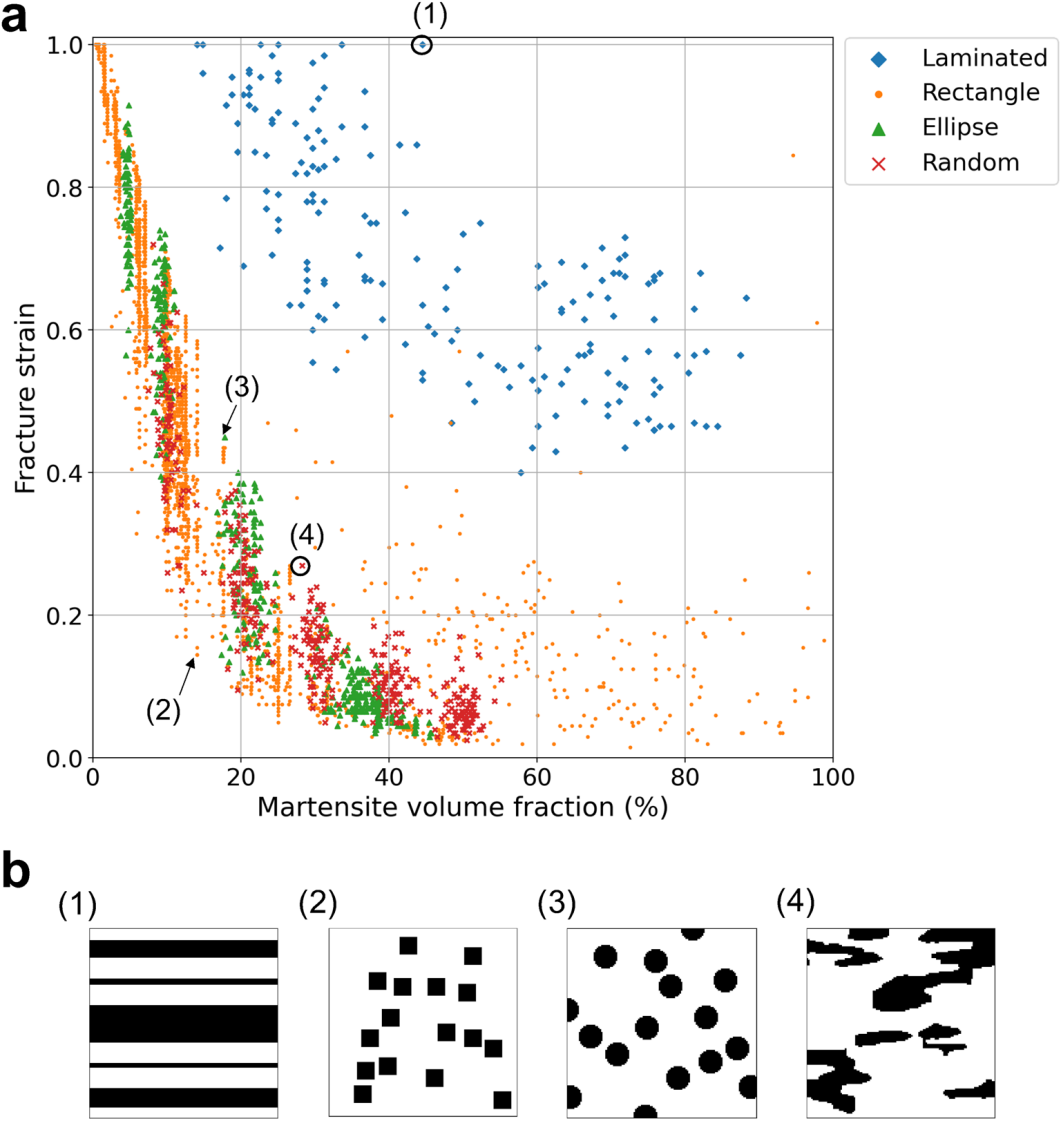
Relationship between martensite volume fraction and fracture strain, and examples of microstructures. (**a**) Plot showing correspondence between martensite volume fraction and fracture strain. (**b**) Examples of microstructures. Their martensite volume fractions and fracture strains are shown in the plot.

For demonstrating the potential of our framework for the structure optimization of multiphase materials in terms of a target property, a simple sample problem is considered. The sample problem is the structure optimization of artificial dual-phase steels composed of the soft phase (ferrite) and hard phase (martensite). Examples of microstructures are shown in Fig. 3. The prepared dual-phase microstructures can be divided into four major categories: laminated microstructures, microstructures composed of rectangle- and ellipse-shaped martensite/ferrite grains, and random microstructures. The size of microstructure images is $128\times 128~\mathrm {pixels}$ and the total number of prepared microstructures is 3824. As an example of a target material property, the fracture strain was selected since fracture behavior is strongly related to the geometry of the two phases. The fracture strain is the elongation of materials at break. As shown in Methodology, the fracture strains for each category were calculated on the basis of the GTN fracture model^18,19^. Figure 4 illustrates the relationship between martensite volume fraction and fracture strain for each category. This shows that laminated microstructures have a relatively high fracture strain. Also, microstructures with a lower martensite volume fraction (higher ferrite volume fraction) possess a higher fracture strain.

**Figure 5:**
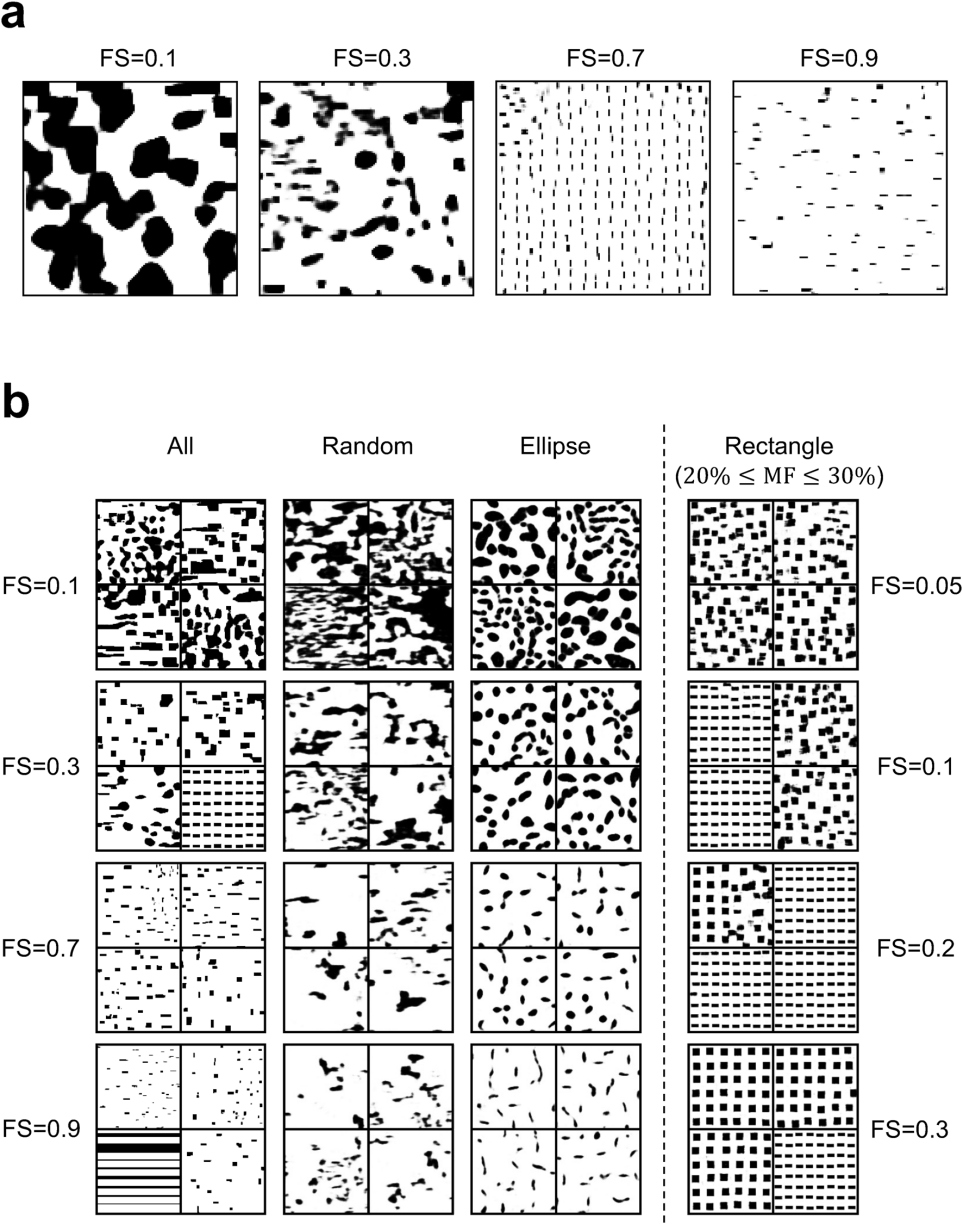
Microstructures generated by the machine learning framework trained by several datasets. (**a**) Examples of microsturctuers generated for several fracture strains by the network trained using All dataset. (**b**) Each column corresponds to the microstructures obtained by the models trained using all microstructures, only the random microstructures, only the microstructures composed of ellipse-shaped martensite grains, or only the microstructures composed of rectangle-shaped martensite grains. However, the Rectangle dataset is limited to include only the microstructures whose martensite volume fraction is between 20% and 30%. The given fracture strains are 0.1, 0.3, 0.7, and 0.9 for the All, Random, and Ellipse datasets, and 0.05, 0.1, 0.2, and 0.3 for the Rectangle dataset.

To show the applicability of our framework, we prepared several datasets: all microstructures (All), only random microstructures (Random), only microstructures composed of ellipse-shaped martensite grains (Ellipse), and only microstructures composed of rectangle-shaped martensite grains (Rectangle). Then, we trained VQVAE and PixelCNN using these datasets. The Rectangle dataset is limited to include only the microstructures whose martensite volume fraction is between 20% and 30% to consider the case in which martensite grains are located separately from each other.

Figure 5a shows examples of microstructures generated for several fracture strains using the network trained by All dataset. Figure 5b summarizes the trend of the microstructures obtained by the networks trained using the above datasets with gradually increasing fracture strain. For the All, Random, and Ellipse datasets, we can see the trend that martensite grains become smaller and thinner as the target fracture strain increases. Since the larger area fraction of the soft phase (ferrite) contributes to the realization of higher elongation as we can see in Fig. 4, this result is reasonable. In addition, it should be noted that the laminated structure corresponding to the highest fracture strain ($\text {FS}=0.9$) was generated only for the All case in which the laminated structures are included in the training dataset. Additionally, in the case of the controlled martensite volume fraction of the input microstructures (Rectangle), the martensite grains tend to arrange more uniformly as the given fracture strain increases.

**Figure 6:**
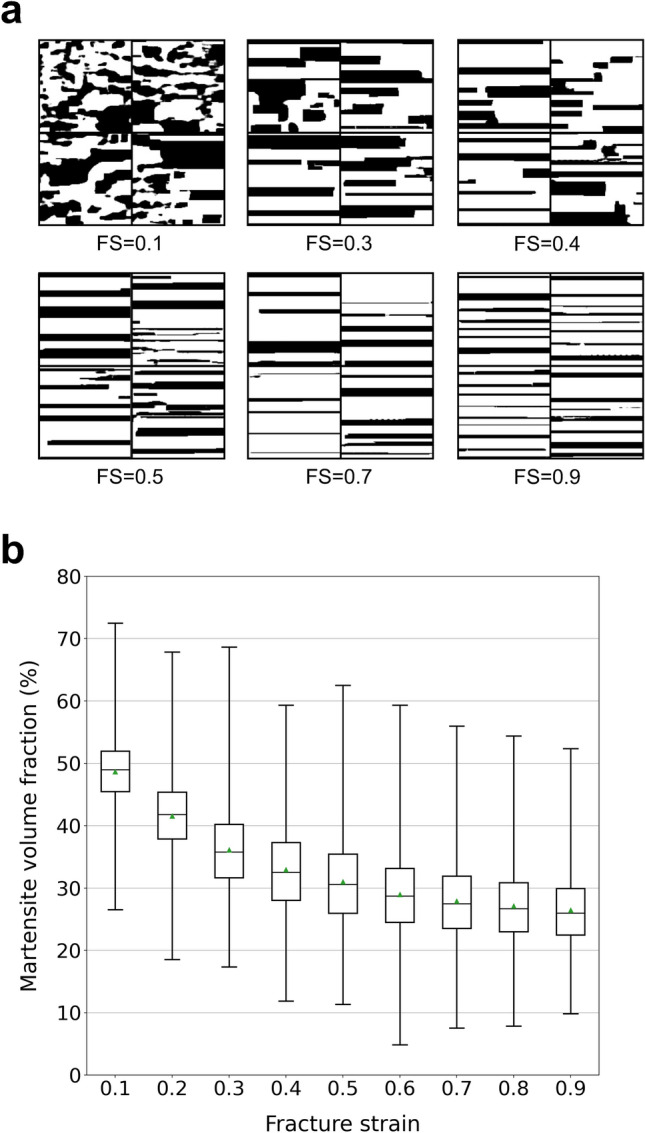
Generated microstructures and trend of martensite volume fraction. (**a**) Microstructures generated at fixed tensile strength and several fracture strains. The tensile strength is set as 700 MPa. The given FSs are 0.1, 0.3, 0.4, 0.5, 0.7, and 0.9. (**b**) Trend of martensite volume fraction relative to the change in fracture strain. For each fracture strain, the martensite volume fractions of 3000 microstructures generated corresponding to the fracture strain and fixed tensile strength ($700\ \mathrm {MPa}$) were calculated. The black lines and green triangles in the boxes denote median and mean values, respectively.

From these results, we can conclude that there are at least two different strategies for the realization of a higher fracture strain: one is to decrease the size of martensite grains and also to arrange them uniformly, and the other to alternatively make a completely laminated composite structure^27^. The fact that the laminated structures never appear without providing the laminated structures in the training dataset implies that there exists an impenetrable wall for a simple optimization process, such as a gradient descent algorithm used to train neural networks, to figure out the robustness of laminated structures from the other structures.

Next, the tensile strength is given in addition to the fracture strain as another label for PixelCNN for considering the balance between strength and fracture strain (ductility). In this case, all microstructure data are used for training. The microstructures are generated at the fixed tensile strength of $700\ \mathrm {MPa}$. The generated microstructures are shown in Fig. 6a. The laminated structures seem to be dominantly selected as the target fracture strain increases. The trend that martensite grains become smaller and thinner is not seen when the tensile strength is fixed.

In addition, the martensite volume fractions were calculated for 3000 microstructures generated corresponding to several fracture strains. The tensile strength was fixed at $700\ \mathrm {MPa}$ again. The box plot of the trend of the martensite volume fraction relative to the change in fracture strain is shown in Fig. 6b. The martensite volume fraction decreases as the given fracture strain increases. At the same time, the martensite volume fraction approaches a constant value. For the realization of a higher ductility without decreasing the tensile strength, the shape of martensite grains approaches that of the laminated structures as the martensite volume fraction decreases. This result implies that laminated structures can achieve a higher tensile strength with a smaller martensite volume fraction. As a result, the laminated structures can be considered as the optimized structures with respect to the shape of martensite grains for the realization of a higher ductility without decreasing their strength. The laminated structures were actually reported to exhibit improved combinations of strength and ductility^27^.

**Figure 7:**
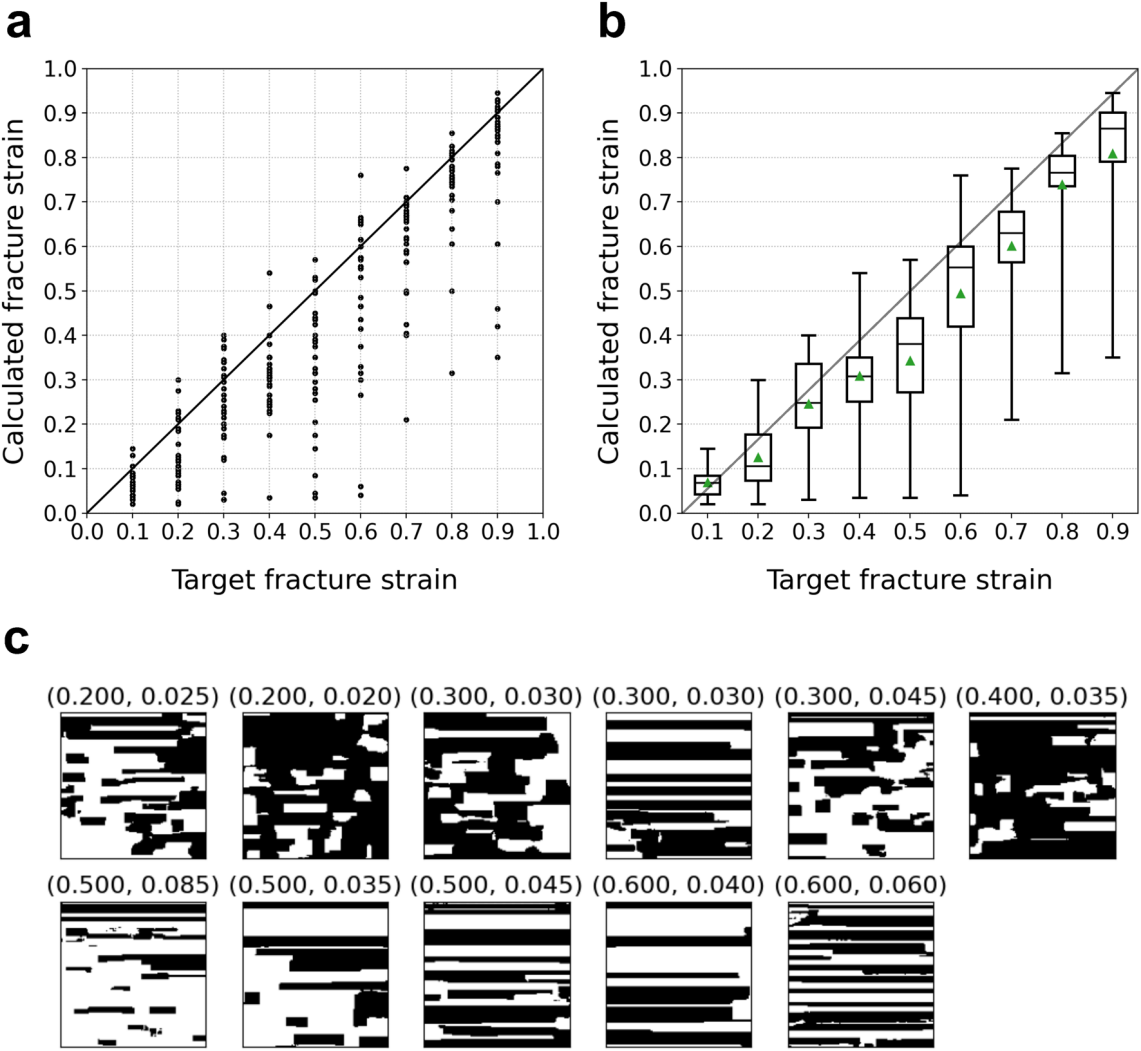
Correspondence between the target fracture strains given as inputs and the actual fracture strains. For each target fracture strain, 30 microstructures were generated. Then, fracture strains are calculated using the physical model^18,19^. (**a**) Plot of relationship. (**b**) Box plot of relationship. The black lines and green triangles in the boxes denote median and mean values, respectively. (**c**) Microstructures whose fracture strains are smaller than 20% of the target fracture strains. The values above the panels denote the given target fracture strains (left) and actual fracture strains (right).

To validate the effectiveness of the present framework, fracture strains are calculated using the physical model^18,19^ for each microstructure obtained using the framework. In this case, the network trained by giving only fracture strain as the target property is used. Figure 7a,b show the correspondence between the target fracture strains for generated microstructures and the actual calculated fracture strains. Also, the coefficient of determination was 0.672. It is clear that our framework captures well the general trend of microstructures relative to the fracture strain. However, it should be noted also that there exist several microstructures whose actual fracture strains are far less than the target strains. Figure 7c shows the typical microstructures whose fracture strains are smaller than 20% of the target fracture strains. Additionally, the coefficient of determination for the data without data points corresponding to the microstructures shown in Fig. 7c was 0.76. All of them are partially incomplete laminated structures. This can be understood as follows. Although laminated structures has a potential to realize higher fracture strains as shown in Fig. 4, this is true only when the microstructures are completely laminated. Even when one martensite layer has a tiny hole, the gap between martensite grains becomes the hot spot that induces much earlier rupture. Thus, the box plot shown in Fig. 7b is understood to show decreasing values as a result of an attempt to completely laminate the structures to realize the given target fracture strain. This indicates that the framework recognizes the structures shown in Fig. 7c to be structurally close to completely laminated structures even though they have far less fracture strains than the completely laminated structures.

As a consequence, these results illustrate that our framework provides a powerful tool for the optimization of material microstructures in terms of target properties, or at least for capturing the trend of microstructures in terms of the change in target property in various cases.

### Identification of microstructures critically affecting material properties

The above results of the generation of material structures corresponding to the target fracture strain indicate that our framework captures the implicit correlation between the material microstructures and the fracture strain. However, generally, it is difficult to interpret implicit knowledge captured by machine learning methods. For that reason, we cannot hastily conclude that machine learning can understand this problem and acquire meaningful knowledge for material design similarly to humans or that it just obtains physically meaningless problem-specific knowledge. Usually, human researchers attain the background physics by noting a part or behavior that will affect a target property during numerous trial-and-error experiments. Generally, this process is time-consuming. Accordingly, approaching implicit knowledge obtained by machine learning methods could be beneficial for achieving a more efficient way to extract general knowledge for material design. Thus, we discuss how to approach the physical background behind the implicit knowledge captured by our framework. In particular, we investigate whether the machine learning framework can identify a part of material microstructures that strongly affects a target property in a similar way human experts can predict on the basis of their experiences.

To identify a critical part of microstructures, we consider calculating a derivative of material microstructures with respect to the fracture strain. This is based on the assumption that human experts unconsciously consider the *sensitivity* of material microstructures to a slight change in target property. Accordingly, the following variable $\Delta$ is defined as the derivative:

3 $$\begin{aligned} \Delta :=\frac{\partial D(\mathbb {E}_{P(\theta |\epsilon _f, M_r)}[ \theta ])}{\partial \epsilon _f}, \end{aligned}$$

where $\mathbb {E}_{P(\theta |\epsilon _f, M_r)}[ \theta ]$ is the expectation of a spatial arrangement of fundamental structures $\theta$ according to $P(\theta |\epsilon _f, M_r)$, which is the probability distribution captured by PixelCNN. Here, $M_r$ and $\epsilon _f$ are the reference microstructure under consideration and the calculated fracture strain for the microstructure, respectively. In other words, $\mathbb {E}_{P(\theta |\epsilon _f, M_r)}[ \theta ]$ is the deterministic function of $\epsilon _f$ and $M_r$. In addition, *D* is the CNN-based deterministic decoder function; hence, $\Delta$ has the same pixel size of the input microstructure images.

If the machine learning framework correctly captures the physical correlation between the geometry of the material microstructures and the fracture strain, $\Delta$ is expected to correspond to the areas in $M_r$ that highly affects the determination of the fracture strain even without giving the physical mechanism itself. For numerical calculation, $\Delta$ is approximated as

4 $$\begin{aligned} \Delta \thickapprox \{D(\mathbb {E}_{P(\theta |\epsilon _f+\Delta \epsilon _f, M_r)}[ \theta ])-D(\mathbb {E}_{P(\theta |\epsilon _f, M_r)}[ \theta ])\}/\Delta \epsilon _f, \end{aligned}$$

where $\Delta \epsilon _f$ is the gap of the fracture strain, which is set as 0.01 in this paper. Because it is difficult to compare quantitatively the distribution of this variable with the critical microstructure distributions obtained from the physical model, in this paper, we only discuss the location of crucial parts. Thus, the denominator $\Delta \epsilon _f$ is ignored for the calculation of $\Delta$ in the rest of this paper.

**Figure 8:**
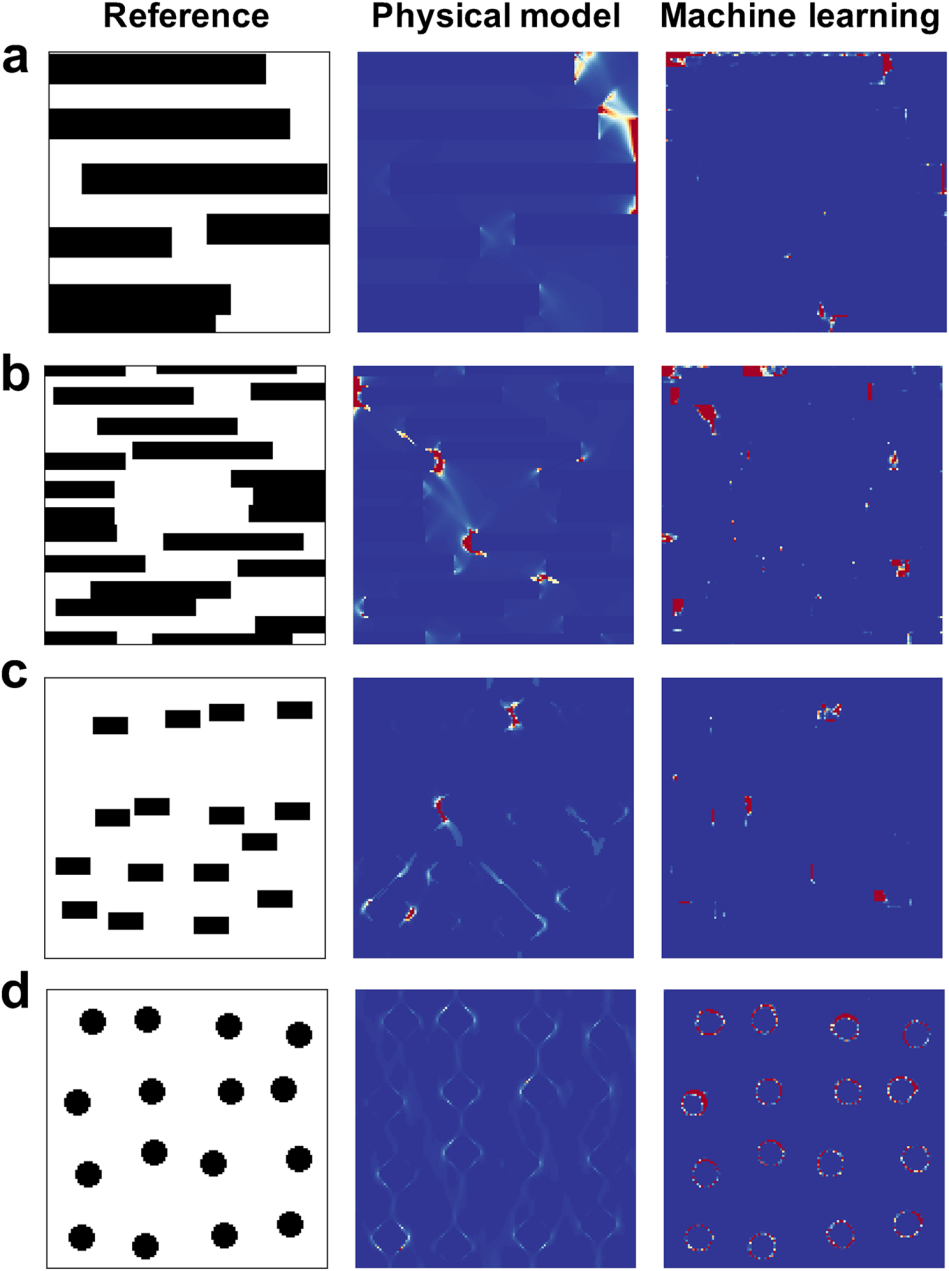
Comparison of derivatives of microstructures with respect to the fracture strain obtained using the machine learning framework with the distributions of void volume fractions calculated on the baisis of physical model. (**a**)–(**d**) Comparisons for several microstructures. The left, middle, and right column correspond to the reference microstructures, the void distributions obtained using the physical model, and the derivative obtained by the machine learning framework, respectively.

Figure 8 shows the comparison of the parts of microstructures critically affecting the determination of the fracture strain obtained by the physical model and our machine learning framework. In the case of the results from machine learning, the absolute values of $\Delta$ defined in Eq. (3) for each pixel are shown as colormaps. On the other hand, because the fracture behavior is formulated as damage and void-growth processes in the physical model, the void distribution in a critical state directly shows the critical points for the determination of fracture strain. Thus, in the case of the physical model, the calculated void distribution in a critical state is shown in Fig. 8. The details of the physical model and the experiment for the determination of some parameters are given in Methodology. For ease of comparison, the ranges of visualized values are changed for each image, while the relative relationships among values of each colormap are kept. Thus, we compare the results qualitatively in terms of the distribution of areas having relatively high values in the next paragraph.

Figure 8a,b illustrate the crucial parts of the microstructures composed of relatively long and narrow rectangle-shaped martensite grains. We can see an acceptable agreement between the results of the physical and machine learning methods in terms of the overall distribution of crucial areas which are shown in red in the colormaps of Fig. 8. In addition, Fig. 8c,d show the parts that critically influence the fracture behavior in the microstructures composed of similarly shaped martensite grains. As an important difference between them, in Fig. 8c, the rectangle-shaped martensite grains are irregularly arranged and some martensite grains are close to each other, which might critically affect the fracture behavior, whereas in Fig. 8d, circular martensite grains are almost regularly arranged. About Fig. 8c, the machine learning framework seems to capture the crucial parts that are predicted by the physical model. As mentioned above, the distributions seem to be dominantly affected by the martensite grains being close to each other. In other words, the short-range interactions among a small number of martensite grains are dominant for the determination of the fracture strain in this case. Also, in Fig. 8d, both the physical model and the machine learning framework can predict that the crucial parts are uniformly distributed in square areas.

**Figure 9:**
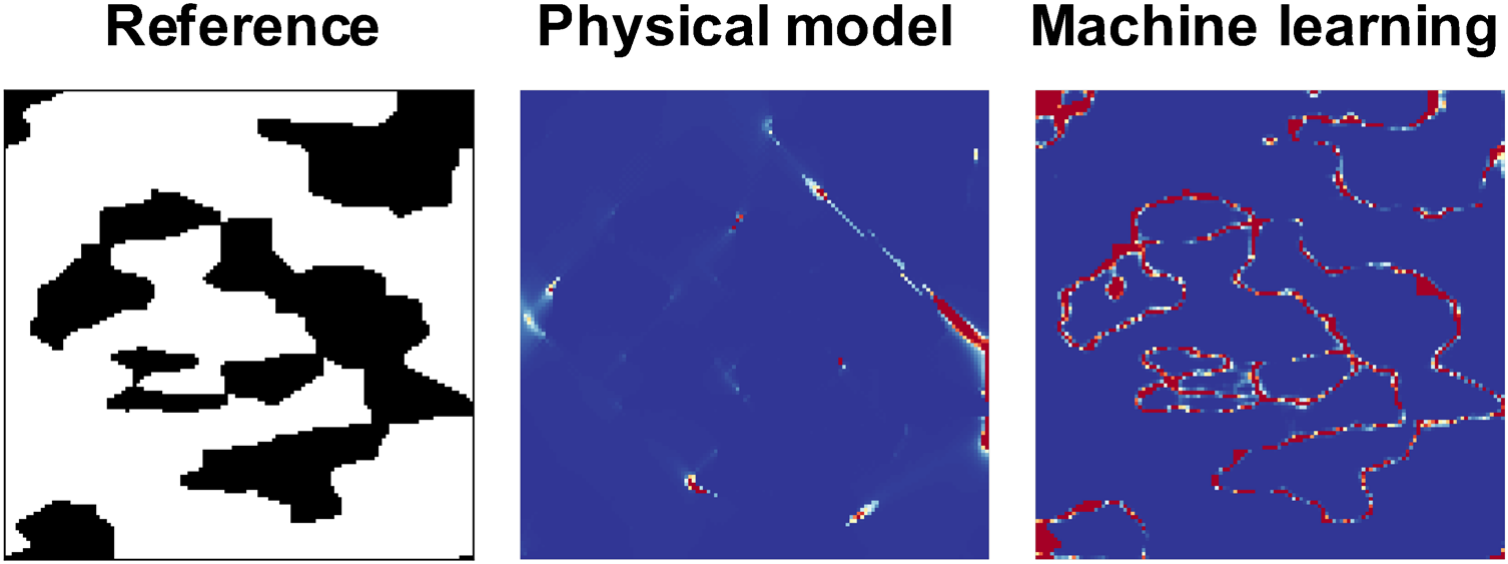
Sample case showing that a relatively long-range interactions among martensite grains are important for the determination of fracture strain.

On the other hand, the physical model also predicts the influence of long-range interactions among martensite grains on fracture behavior, which can be seen in Fig. 8c,d as a bandlike distribution. However, the bandlike distribution resulting from the long-range interactions does not seem to be captured by the machine learning framework owing to the characteristic of PixelCNN. Because a global stochastic relationship among the fundamental elements is factorized as a product of stochastic local interactions in PixelCNN as defined in Eq. (1), the extent of interaction exponentially decreases as distance increases. Therefore, the long-range interactions are difficult to be captured by PixelCNN. The discussion of the limitation of PixelCNN in capturing long-range interactions and a remedy for this limitation can be found in^28^. Figure 9 illustrates a sample case showing that the relatively long-range interactions are important for the dertermination of fracture strain. In this case, the determination of the part that critically affects the fracture behavior seems to be difficult using the framework based on PixelCNN.

For incompletely laminated structures such as that shown in Fig. 8a, the martensite layers are expanded to achieve a higher fracture strain even though increasing the martensite volume fraction basically contributes to the decrease in the fracture strain, as shown in Fig. 4. Similarly, we can see in Fig. 8c that the martensite grains tended to expand to fill the hot spots between them. Additionally, as mentioned above, even though completely laminated structures are structurally similar to incompletely laminated structures, the fracture strains of completely laminated structures are much higher than those of incompletely laminated structures. Thus, eliminating tiny holes that could be causes of hot spots and reaching ${ completely}$ laminated structures markedly improve their fracture strains. Altogether, these results imply that the framework recognizes the potential of laminated structures to achieve a higher fracture strain in a similar way that human researchers reach an intuition on completely laminated structures as a result of the consideration of reducing the occurrence of hot spots.

From the above results, we can conclude that our framework can identify the areas that critically affect a target property without human prior knowledge when the local topology of microstructures is dominant for the target property. This implies that machine learning designed consistent with metallurgists’ process of thinking can approach the background or the meaning of the implicitly extracted knowledge in a similar way that humans acquire an empirical knowledge.

## Conclusion

The machine learning framework composed of VQVAE and PixelCNN is presented as a tool for reflecting metallurgists’ track of thought for interpreting and developing material structures as a computational framework. To show the performance of the present approach, an optimization problem of artificial dual-phase metallic materials concerning a fracture property was analyzed. The results indicate that the framework clearly captures the trend of material microstructures with respect to the change in the target properties, such as fracture elongation and strength to elongation balance, and thus, provides a powerful tool to optimize microstructure for a target property.

The physical background of the implicit knowledge captured by the present framework was further investigated. In particular, the ability of identifying a part of microstructures critically affecting the target physical property without giving an explicit physical mechanism itself was examined. Results show that the distribution of the hot spot can be identified in a similar way that human experts intuitionally recognize a sensitive part of microstructures to change of the property based on their experiences. Thus, this methodology provides a efficient data-driven way to attain empirical knowledge for material design which are usually obtained by human researchers’ trial end error process. In conclusion, this paper not only demonstrates the potential of our framework to analyze the structure-property relation for optimization of material structures but also shows that to imitate human experts’ train of thought could be a guide for approaching a background of the implicit knowledge captured by machine learning.

## Data Availability

Original microstructure data are available from the corresponding author upon reasonable request.

## Code availability

The sample code is available on https://github.com/inouejunyalab/for_public/tree/main/VQVAE_PixelCNN.
